# Perceptual Temporal Asymmetry Associated with Distinct ON and OFF Responses to Time-Varying Sounds with Rising versus Falling Intensity: A Magnetoencephalography Study

**DOI:** 10.3390/brainsci6030027

**Published:** 2016-08-05

**Authors:** Yang Zhang, Bing Cheng, Tess K. Koerner, Robert S. Schlauch, Keita Tanaka, Masaki Kawakatsu, Iku Nemoto, Toshiaki Imada

**Affiliations:** 1 Department of Speech-Language-Hearing Sciences, University of Minnesota, Minneapolis, MN 55455, USA; koern030@umn.edu (T.K.K.); schla001@umn.edu (R.S.S.); 2 Center for Neurobehavioral Development, University of Minnesota, Minneapolis, MN 55455, USA; 3 Speech-Language-Hearing Center, School of Foreign Languages, Shanghai Jiao Tong University, Shanghai 200240, China; 4 English Department & Institute for Language, Cognition and Brain Sciences, School of Foreign Studies, Xi’an Jiaotong University, Xi’an, Shaanxi 710049, China; 5 School of Science and Engineering, Tokyo Denki University, Ishizaka, Hatoyama, Hiki-gun, Saitama, 350-0394 Japan; ktanaka@mail.dendai.ac.jp; 6 School of Information Environment, Tokyo Denki University, 2-1200, Muzai-gakuendai, Inzai-shi, Chiba 270-1382, Japan; kawakatu@asrl.dendai.ac.jp (M.K.); nemoto@sie.dendai.ac.jp (I.N.); 7 Institute for Learning and Brain Sciences, University of Washington, Seattle, Washington 98195, USA; imada@u.washington.edu

**Keywords:** MEG, auditory ON response, auditory OFF response, equivalent current dipole (ECD), minimum norm estimation (MNE), phase locking factor (PLF), temporal asymmetry index (TAI)

## Abstract

This magnetoencephalography (MEG) study investigated evoked ON and OFF responses to ramped and damped sounds in normal-hearing human adults. Two pairs of stimuli that differed in spectral complexity were used in a passive listening task; each pair contained identical acoustical properties except for the intensity envelope. Behavioral duration judgment was conducted in separate sessions, which replicated the perceptual bias in favour of the ramped sounds and the effect of spectral complexity on perceived duration asymmetry. MEG results showed similar cortical sites for the ON and OFF responses. There was a dominant ON response with stronger phase-locking factor (PLF) in the alpha (8–14 Hz) and theta (4–8 Hz) bands for the damped sounds. In contrast, the OFF response for sounds with rising intensity was associated with stronger PLF in the gamma band (30–70 Hz). Exploratory correlation analysis showed that the OFF response in the left auditory cortex was a good predictor of the perceived temporal asymmetry for the spectrally simpler pair. The results indicate distinct asymmetry in ON and OFF responses and neural oscillation patterns associated with the dynamic intensity changes, which provides important preliminary data for future studies to examine how the auditory system develops such an asymmetry as a function of age and learning experience and whether the absence of asymmetry or abnormal ON and OFF responses can be taken as a biomarker for certain neurological conditions associated with auditory processing deficits.

## 1. Introduction

One fundamental property of the human auditory system is to automatically detect and respond to sound sources and changes. Rising and falling intensities are two basic dynamic patterns in the acoustic environment. A damped sound is characterized by the abrupt occurrence of high-intensity onset and a gradual fade-out whereas a ramped sound has the opposite pattern. Behavioral research has shown a perceptual bias for rising-intensity (or ramped) sounds. Compared with the time-reversed stimuli, listeners report hearing different timbre quality with stronger tonality for the ramped tone [1,2] and overestimate the ramped sounds in subjective duration [3,4,5,6,7], overall loudness [5,8,9], and the amount of perceived loudness change within the stimuli [10,11,12]. For instance, Schlauch et al. (2001) found that ramped sounds ranging from 10 to 200 ms were perceived to be longer than the time-reversed damped sounds, which had identical physical duration as well as long-term spectral power and envelope spectra. This overestimation was also found for sounds longer than 200 ms [6]. In the literature, the reported bias for ramped sounds is referred to as “auditory perceptual asymmetry” or “perceptual looming”, which is affected by the spectral complexity of the carrier sound for the ramped and damped intensity modulation. When the carrier is spectrally more complex than a sinusoidal tone, the asymmetry decreases or may even disappear [1,3,10,13,14]. 

Presumably, the perceptual asymmetry phenomenon arises from distinct neural coding of the two dynamic intensity patterns, which may (or may not) require attentional processing and learning experience. One ecologically motivated explanation points to the attentional system of adaptive alertness, which is associated with auditory motion perception of approaching versus receding sound sources [14,15,16]. Two psychophysical accounts have also been proposed [3,7,8]. One is that listeners can perceptually separate the attack and gradual release of a damped sound, ignoring a segment of the decay portion as “echo” and thereby perceiving a shorter and softer sound. Another is that the abrupt offset of ramped sounds may produce stronger and persistent activity, which results in overestimation of duration and loudness. Digiovanni and Schlauch (2007) studied both of these possible mechanisms [7]. They measured subjective durations in two groups of listeners. One group was instructed to match the durations of the ramped and damped sounds without any special instructions (null instructions) whereas the other group was instructed to “include all aspects of the sounds”. The two different instruction sets produced very different results. When no special instructions were offered, the ramped sounds were judged to be 50%–90% longer than the damped sounds. By contrast, the group told to include all aspects of the sounds judged the ramped sound to be between 10% and 20% longer than damped sounds. The larger perceptual asymmetry for the null instruction set is believed to be a result of subjects ignoring the echo of the damped sounds. The remaining perceptual asymmetry (10%–20%), when subjects were told to include all aspects of the sounds, is attributed to persistence of excitation. Temporal masking patterns were measured and the temporal extent of the excitation (9%–24% longer for ramped than for damped sounds), provided further support for the persistence explanation. That is, temporal footprint representing the range of durations over which a ramped or damped sound used as a masker could interfere with detection of a signal was longer for ramped sounds than for damped ones, and that difference corresponds to the perceptual judgments of duration when subjects are asked to attend to all aspects of the sounds. Ries et al. (2008) reported a nearly identical finding for temporal masking patterns [5]. 

Although the behavioural temporal asymmetry is well established, the neural mechanisms are not completely understood. Animal neurophysiology work has demonstrated asymmetric neural representations for ramped and damped sounds at multiple processing sites in the auditory pathway [17,18,19]. There is evidence that persistence of excitation after the stimulation offset is longer for the ramped sound than that of the damped sound [20]. Stronger gamma (45–90 Hz) activities were also found for the ramped auditory signals in comparison with damped sounds [21]. It has been suggested that the ON-neurons in the auditory cortex may play an important role in detecting sound source movement [22] whereas the OFF-neurons enable more precise temporal coding of the intensity envelope [17,23]. Unlike the animal studies, human research has primarily relied on experimental paradigms that require attentive tasks. In earlier work [13,24], a peripheral mechanism of input suppression was proposed. However, the peripheral model was found to be not able to address the auditory bias for sounds longer than 50 ms. There is a known asymmetric coding that differences in temporal onsets are encoded more readily and accurately than differences in offsets for sounds of various lengths [25]. A plausible account would require detailed knowledge about neural coding of the ramped and damped sounds in the whole auditory pathway [6,24], especially at the cortical level. 

Auditory stimulation in event-related paradigms typically produces a robust ON response (also known as the auditory N1) in adult listeners irrespective of the listener’s attention. A similar off-N1 response can be observed at the cessation of an auditory stimulus longer than 100 ms. Both the ON and OFF responses have been considered to represent similar automatic cortical responses to abrupt changes due to their similar properties in latency, topography, and source localization [26,27,28,29]. While the evoked ON and OFF responses are well documented in electroencephalography (EEG) and MEG studies [25,26,27,28,30,31,32,33,34,35], there has been a lack of human neurophysiological data that systematically examined the asymmetric ON and OFF responses for ramped and damped sounds. Findings and interpretations are mixed. Several studies showed that perceived duration was not a simple reflection of the differences between the ON and OFF responses [3,36,37]. Intracellular recordings from the primary auditory cortex in animals [38] as well as from the left auditory cortex in humans [39] indicated that the OFF response neurons played a more important role in sound duration perception than the ON response neurons. In contrast, several MEG studies reported that the ON response was a good predictor for perceived differences such as hissiness or pitch salience of ramped and damped stimuli [40,41,42]. However, these MEG studies used a ramped/damped envelope shorter than 50 ms and thus did not provide OFF response measures in relation to the perceived temporal asymmetry. 

The primary goal of the present study was to compare evoked ON and OFF responses to ramped and damped auditory stimuli in terms of response amplitude, latency and source localization and explore possible brain-behavior correlations for the temporal asymmetry phenomenon. To replicate the perceptual temporal asymmetry, we conducted behavioural tests of duration comparison and estimation. We hypothesized that the ON and OFF responses would show differences in amplitude, latency measures and source localization. In particular, damped sounds would produce a larger and earlier ON response but a smaller OFF response relative to the ramped sounds, which could provide a good opportunity to verify the two explanations regarding persistence of excitation for ramped sounds and ignoring the echo of the damped sounds. Given that the OFF response is also sensitive to the intensity level and rise/fall-time of the auditory stimuli [43,44], we predicted that the ON and OFF response amplitude measures might show good correspondence with the behavioral data [39]. 

The secondary goal was to examine how spectral complexity of the auditory stimuli might influence perceived temporal asymmetry and the neural measures of ON and OFF responses. We used two pairs of stimuli by manipulating the intensity envelope and spectral complexity, one pair based on a sinusoidal tone (S stimuli) and the other based on a complex piano note (C stimuli). Based on previous studies [1,3,10,13,14], we hypothesized that adding spectral complexity to the ramped and damped sounds might reduce the perceptual bias effect due to the additional perceptual attributes and thus weaken the brain-behavior correlational strength. 

The third goal was to examine how the distinct intensity envelopes of the auditory stimuli drove the ON and OFF responses via neural phase locking across trials in the different cortical oscillatory frequency bands. Cortical oscillation rhythms reflect properties of large-scale neuronal population excitability and discharge synchronization/desynchronization that subserve various perceptual, attentional, and integrative functions [45,46,47]. The phase locking measure (or inter-trial phase coherence) indicates the degree of consistency in temporal alignment of neural responses to the stimulus/task characteristics. In the auditory domain, neural oscillatory activities are thought to reflect different aspects of sound processing. Theta (4–8 Hz) activity has been associated with processing the temporal and spectral attributes of spoken sentences, respectively [48,49]. Theta and alpha (8–14 Hz) bands are also sensitive to the rise in time of the acoustic stimulus onset [50,51]. We hypothesized that in comparison with ramped stimuli, the larger and earlier ON response for damped sounds would be associated with enhanced phase locking in theta and alpha activities. We were also interested in testing whether the ramped stimuli would induce stronger gamma activity in the OFF response, which was shown in an earlier animal study [21]. 

## 2. Materials and Methods 

### 2.1. Participants

Six right-handed male adults with normal hearing (thresholds < 25 dB HL for pure tones in the range of 250–8000 Hz).participated in the study (24–38 in age). They were recruited after screening for hearing, handedness, and auditory evoked responses. A steady 1 kHz tone (200 ms in duration) served as a reference stimulus to test the robustness of the auditory ON and OFF responses in each subject. For the current study, we only included normal-hearing subjects who participated in previous auditory MEG experiments in the same lab and showed clear N1m response with bilateral dipole activity. None of the subjects had medical history of speech, language, or hearing disorders. Informed consent was obtained from each volunteer subject in accordance with approvals from the Institutional Review Boards at the University of Minnesota and Tokyo Denki University (Ethic approval code: 0605M85808).

### 2.2. Stimuli

**Figure 1:**
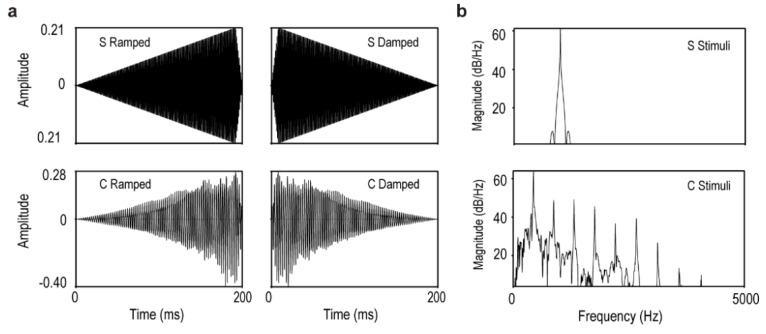
Acoustic representations of the S and C ramped and damped stimuli. (**a**) Sound waveforms; (**b**) Power density spectra.

The ramped and damped stimuli consisted of two pairs of sounds (Figure 1). The two sets of stimuli were 200 ms in physical duration. The first pair of ramped and damped sounds (S stimuli) was based on a 1000 Hz sine wave tone. A linear fade-in envelope was applied to the simple tone in making the ramped sound, and the damped sound was its time reversal. This process ensured that the physical duration, intensity, and spectral contents were identical for the ramped and damped sounds. The second pair of ramped and damped sounds (C stimuli) was based on a synthesized piano note with a fundamental frequency of 440 Hz. The piano note was first synthesized in GuitarPro5 (Arobas Music, Lille, France). It had a nonlinear falling intensity envelope. Time reversal was applied for the ramped counterpart. To verify the existence of ON and OFF responses within each subject, we used a 200 ms long reference tone at 1000 Hz with a steady intensity envelope with a rise/fall time of 10 ms. The same 10 ms rise time treatment was applied to the onset of the damped stimuli and the 10 ms fall time to the offset of the ramped stimuli to avoid the “click” percept associated with transient distortion. All sounds were normalized to have the same RMS (root mean square) average intensity. 

### 2.3. MEG Recording 

The MEG experiment used a whole-scalp 122-channel neuromagnetometer system (Neuromag-122, Neuromag Ltd., Helsinki, Finland) in a magnetically shielded room at the Research Center for Advanced Technologies, Tokyo Denki University, Japan. The Neuromag-122 featured an inherent device coordinate system with 122 sensors at 61 sites covering the whole head. The MEG recording procedure was completely non-invasive. Prior to the MEG experiment, the subjects were taken to an MRI facility in the same research center (Stratis II, a 1.5 T Superconductive Magnetic Resonance Imaging System, Hitachi Co., Tokyo, Japan) for structural brain imaging. The MRI protocol parameters (TR = 36 ms, FA = 40 degrees, TE = 8.9 ms, NEX = 1.0) for the echo sequence were the same as in previous MEG publications [52,53]. The number of slices were 193 with image slice thickness at 1 mm. The MRIs allowed the construction of realistic head models for each individual subject to improve the precision of source localization. 

During MEG recording, the subjects were seated in a nonmagnetic chair inside the magnetically shielded room. The subject’s head position in the MEG device was monitored using four head position indicator (HPI) coils in reference to the spatial coordinate frame defined by the nasion, the left and right preauricular points relative to the individual’s MRI head model. The positions of the HPI coils with respect to the anatomical landmarks were first measured with a three-dimensional Polhemus Isotrak digitizer outside the shielded room. When the subjects were seated under the MEG dewar, HPIs were measured to ensure that the positioning accuracy was within 98%–100% for each coil. Vertical electro-oculograms (EOGs) were recorded online with a pair of bipolar electrodes pasted at the supraorbital and infraorbital ridge of the right eye. The impedances of the bipolar electrodes were lower than 5 kOhm. The stimuli were binaurally delivered at a sensation level of 50 dB via non-magnetic foam earplugs through a non-echoic plastic tube system. The sensation level was individually calibrated for each subject using the steady 1 kHz reference tone with its mean RMS (root mean square) level matched to that of the ramped and damped stimuli. Specifically, the binaural hearing threshold for the 1 kHz steady tone was determined for each individual subject at the beginning of the MEG session. Subjects were asked to verify that they could hear the acoustic stimuli clearly at a comfortable level before proceeding to MEG recording. This stimulus presentation method based on individually calibrated audiometric sensation level has previously been applied in our hearing research studies [54,55] and other MEG studies [50,56].

A commonly used passive listening paradigm was adopted with a distraction task to help minimize potential differences due to preferential listening [57]. Stimulus presentation used an alternating short block design. There were five different stimuli, including the ramped and damped sounds in simple and complex conditions and the reference tone. Each short block consisted of 20 identical stimuli with an interstimulus interval randomized in the range of 1000–1100 ms. The inter-block interval was 5 s. No two identical stimulus blocks were presented consecutively. A similar alternating block design had been used in a previous study [58]. In the present study, the subjects were instructed to watch a self-chosen movie projected to a white screen, which was placed approximately 1.5 m in front of the subject. During the experiment, the subjects were asked to concentrate on the muted movie with subtitles and ignore the auditory stimuli. The MEG signals were bandpass-filtered from 0.03 to 100 Hz and digitized at 497 Hz. Epochs with amplitude greater than 3000 fT/cm or EOG greater than 150 μV were rejected to exclude data with blinking and movement-related artifacts or other noise contamination. For each subject, at least 80 good epochs were averaged for each stimulus as in our previous MEG publications [52,53].

### 2.4. Global Field Power Analysis

To verify the experimental hypotheses, we conducted global field power analysis as well as two source localization methods [59]. In the waveform analysis, the raw MEG data were digitally low-pass filtered at 40 Hz and corrected with a pre-stimulus baseline of 100 ms. As our interest was in the neural coding of the physical stimulus features instead of neural sensitivity in discriminatory responses, the first stimulus in each block was omitted from averaging to avoid possible elicitation of a mismatch field response to the previous block of stimuli. MEG waveform amplitude was defined as the vector sum of amplitudes at two orthogonal channels in the same sensor location. To derive a composite measure of differences in MEG activity for the averaged ON and OFF responses for the ramped and damped stimuli, we analyzed the global field power (GFP) for each stimulus and each subject. Similar to the calculation of GFP for EEG signals (e.g., [54]), the GFP is the root mean square of magnetic fields across all the 61 recording sensor sites at each time sample. The GFP measure has been shown to be an objective and reliable quantification method independent of sensor selection in EEG and MEG studies [60,61,62]. 

### 2.5. Source Localization Analysis

To localize the ON and OFF MEG components, we employed distributed source analysis using minimum norm estimation (MNE). The MNE approach is thought to be more appropriate when the distribution of source activity is poorly known [63]. The MNE analysis procedure here was performed using the MNE-Suite by Dr. Matti Hämäläinen, including the following steps:

(1) Head model preparation. The individual subject’s MRIs were converted and processed using the Freesurfer software to derive the boundary element models.
(2) MRI-MEG co-registration. After stimulus set operations were performed for filtering, artifact rejection and averaging, the MEG waveform data were loaded for head position adjustment relative to each subject’s Head Position Indicator (HPI) data and the 3-D locations of the nasion, left and right preauricular points. We used four HPI coils in the experiment; two were positioned in the forehead, and the other two were right behind the two ears.
(3) Standardized forward and inverse solution. Forward and inverse solutions were derived following the recommended settings in the MNE-Suite. Baseline noise covariance matrix was calculated for each averaged data set. To perform MNE averaging across subjects and regions of interest analysis, the individual MNE data were morphed to a standard brain model.
(4) Regions of Interest (ROI) analysis. Based on the brain activation patterns, two ROIs in the standard brain space were chosen, namely, the superior temporal (ST) and inferior parietal (IP). The ROIs were anatomically defined and annotated with corresponding Talairach coordinates in the MNE-Suite. The individual MNE waveforms for each ROI were exported and further analyzed in Matlab.
(5) MNE movie generation. For visualization purposes, the grand mean results for each stimulus were exported as movie files with MNE results expressed in dSPM (dynamic statistical parametric mapping) values integrated at every 10 ms [64,65]. The movie frames with peak activities were selected in the two post-stimulus windows, 70–150 ms and 250–350 ms, and plotted to illustrate the spatial localization of the ON and OFF responses.

To help determine whether the localization results were similar for the ON and OFF responses in each direction of the x, y, and z head coordinates, we also applied the single moving equivalent current dipole (ECD) model in each hemisphere [63]. Grand mean isofield contour map data for the auditory ON and OFF responses were extracted with BESA (Version 6.0, MEGIS Software GmbH, Gräfelfing, Germany). ECD modelling is often adopted in the analysis of auditory evoked responses, which predominantly show dipolar field distributions. In the ECD approach, an optimal solution is sought in the calculation of the dipole parameters in terms of location, amplitude, and direction of the source current by minimizing the least-square error between the measured and predicted signals. However, the point-like ECD source solution might be an oversimplification as the auditory N1 activity has been shown to contain several subcomponents with contributions from temporal, parietal and frontal regions in both hemispheres [30,66,67,68,69]. In contrast, the distributed source MNE model not only shows the spread of activity but also allows visualization of simultaneous activities at multiple sites as well as the strength of activity at different vertices within a specified region of interest. While the ECD solutions are restrictive in the number of active point sources without any information about the extent of activation, the MNE results tend to be biased towards superficial sources without properly measuring the depth information [70]. In this regard, we combined both point source and distributed source modelling in order to take advantage of their complimentary features to explain the cortical source activities of the ON and OFF responses.

### 2.6. Phase-Locking Factor Analysis

Phase-locking factor (PLF) in gamma (30–70 Hz), alpha (8–14 Hz), and theta (4–8 Hz) bands were analyzed for each subject. Phase consistency across trials was computed based on Morlet wavelet analysis at the target frequency bands [47,71,72]. The absolute PLF values in the range of 0 (non-phase-locked activity) and 1 (strictly phase-locked activity) were calculated for the ramped and damped sounds using PLF functions from the Matlab-based 4d Toolbox (version 1.2 developed by Ole Jensen) [72]. The representation of the phase for trial *j*, Φ*_j_*, at a given time (*t*) and frequency (*f*_0_), was derived from the convolution of the complex wavelets defined as Formula (1–3)

*w*(*t*, *f*_0_) = *Aexp* (−*t*^2^/(2σ*_t_*^2^)) *exp* (2*i*π*f*_0_*t*) (1)

where

σ*_t_* = *m/2*π*f*_0_ (2)

*A* = 1/(2πσ*_t_*^2^)^1/2^ (3)

*i* is the imaginary unit, *m* defines the compromise between time resolution and frequency resolution, and *f*_0_ is the center frequency. The wavelet had a Gaussian shape in both the time domain and the frequency domain around its central frequency *f*_0_. The width of Morlet wavelet (see Formula (4)) was set at 7 [47].

(*m* = *f*_0_/σ*_f_*) (4)

The phase for trial *j* was obtained for the signal *s_j_*(*t*)*,* normalized by the amplitude, see Formula (5).

Φ*_j_*(*t*, *f*_0_) = *w*(*t*, *f*_0_) *s_j_*(*t*)/|*w*(*t*, *f*_0_) *s_j_*(*t*)|. (5)

The spectral amplitudes of the wavelets were normalized so that the total energy was 1. The normalized complex time-varying energy of each single trial was averaged across trials for each stimulus. The PLF value over N trials was calculated as Formula (6).

*PLV*(*t*, *f*_0_) = 1/*N* |∑_*j* = 1_^*N*^ Φ*_j_*(*t*,*f*_0_)| (6)

We specifically used a set of wavelets with the frequency (*f*_0_) ranging from 1 to 90 Hz in 1 Hz steps. 

All the PLF computations were conducted on the individual subjects’ trial-by-trial raw data after artifact rejection without applying the 40 Hz lowpass filter. As previous research has shown that OFF response plays a more important role in duration coding [38], the gradiometer sensor that showed the largest OFF responses in MEG waveform analysis was selected for each stimulus and each subject. The mean absolute PLF values were averaged for theta band (4–8 Hz), alpha band (8–14 Hz), and gamma band (30–70 Hz) in a window of 20 ms centering around the ON and OFF peaks for statistical comparisons between ramped and damped sounds. 

### 2.7. Behavioral Tests on Subjective Duration

Right after the passive listening session for MEG recording, two separate active listening tests with 1-min breaks in between were administered using the same stimulus presentation level and ear insert setting to verify the perceptual bias for auditory looming in terms of subjective duration. In Test 1, the subjects were asked to press buttons to indicate whether the two sounds presented in a stimulus-pair trial were equally long. If not, they were further instructed to indicate whether the first or second stimulus sounded longer. The two sounds in a stimulus pair were separated by a 250 ms silence interval, and the inter-trial interval was randomized in the range of 1500–2000 ms. The stimulus presentation order was randomized for the stimulus-pair trials consisting of two stimulus conditions (S and C stimuli), two trial orders (ramped-damped and damped-ramped), and two foil orders (ramped-ramped and damped-damped). Each stimulus pair was presented 20 times. In Test 2, the subjects were asked to perform a duration judgment task with a procedure modified after DiGiovanni and Schlauch (2007) [7]. In this task, the subjects were instructed to include all aspects of the auditory stimuli when making their judgments. In a given trial, the listeners were required to adjust the length of a steady 1 kHz tone to match the duration of the target sound (one of the five sounds for the MEG recordings; four S and C ramped/damped stimuli and the 200 ms steady 1 kHz reference tone). Linear rise/decay of 10 ms was applied to the adjustable steady sound. The adjustable duration range was set at ±80% of the target [6]. The inter-stimulus interval for each matching sound pair (target sound followed by adjustable sound) was 500 ms, and the inter-trial interval was 1500 ms. Trials were presented in random order with each target sound tested 20 times. 

### 2.8. Statistical Analysis

For all behavioral and MEG measures, we conducted statistical tests for normality, variance homogeneity, and outlier detection to ensure no existence of statistical outliers or other variability/normality problems that would cause a violation of the assumptions of the statistical tests. Results from behavioral Test 1 were calculated in terms of percentage of trials for which the ramped sounds were judged to have a longer duration. Response sensitivity in terms of d-prime score were then calculated using signal detection theory [73]. Given the categorical behavioral responses, the Fisher’s Exact Test was applied to each individual subject’s data to verify the subjective duration bias for auditory looming. Magnitude estimation results of behavioral Test 2 were averaged for each target sound for each individual subject for an assessment of the relative strength of perceptual asymmetry for the S and C stimuli. A repeated-measures ANOVA test was conducted for the perceived duration data to verify the significance of the temporal asymmetry phenomenon with the two main factors, stimulus intensity envelope (ramped vs. damped) and stimulus condition (S vs. C stimuli). 

Repeated-measures ANOVA tests were performed on the peak ON and OFF responses for the GFP, ECD, and MNE data, respectively. The main factors of interest included intensity envelope (ramped vs. damped), response type (ON vs. OFF), stimulus condition (S vs. C stimuli), and hemisphere (left vs. right). The hemisphere factor did not apply to the GFP data as the root mean square calculation used for all 122 MEG sensors. To visualize the temporal evolution of significant differences between ramped and damped stimuli, two-tailed point-to-point *t*-tests were conducted over the entire epoch [58,74]. In order for an interval to be considered significantly different between the two sounds, at least eight consecutive points (approximately 16 ms) needed to reach the significance level of 0.01 [52,58,75]. 

To examine brain-behavior correlates, we adopted the temporal asymmetry index (TAI) formula for both behavioral and brain measures (see Formula (7)) [24,40,76],

(Q_ramped_ − Q_damped_)/(Q_ramped_ + Q_damped_) (7)

where Q stands for measures of the same type from an individual subject. In calculating the neural TAI measures, we specifically looked at the ON and OFF responses in the left and right hemispheres separately in terms of ECD amplitude and latency data. To confirm the correlation analysis with the ECD data, TAI measures were also derived from MNE amplitude and latency data in the superior temporal region. We were particularly interested in finding out which MEG response measure would be a better predictor of perceived duration asymmetry and which source localization method provided stronger correlation results. As the linear assumption in Pearson’s correlation is likely problematic for the TAI measures for brain and behavioral responses, we adopted the Spearman rank correlation that assumed a monotonic but not necessarily linear relationship [77]. Considering the sample size, we used the Spearman test function in a new Matlab toolbox for robust correlation analysis, which included a resampling bootstrap procedure to verify the significance of the correlation coefficient obtained from the small sample [78]. We adopted false discovery rate (FDR) with the Benjamini-Hochberg procedure [79] for correcting the *p*-values in multiple comparisons. 

**Figure A1:**
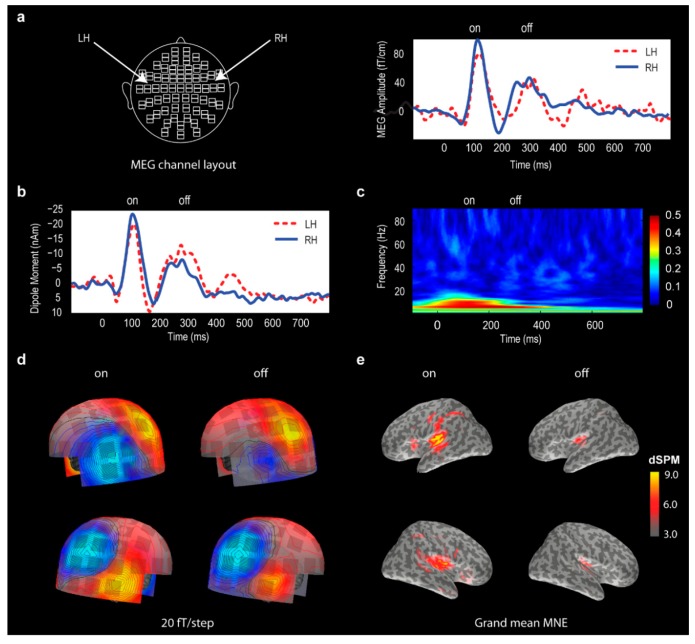
MEG data with clear ON and OFF response patterns for the steady 1 kHz reference tone. (**a**) 122 channel layout and grand mean MEG waveforms from selected sensors covering the left (LH) and right (RH) hemispheres; (**b**) Grand mean equivalent current dipole waveforms in LH and RH; (**c**) Grand mean phase locking factor plot as a function of time; (**d**) Representative isofield maps (derived with MNE-suite from one individual subject) for the ON and OFF responses; (**e**) Grand mean MNE activities projected to the standard brain for ON and OFF responses corresponding to the ON and OFF peak latencies in GFP analysis. A 10 ms integration window was used in generating the MNE activation maps.

**Figure A2:**
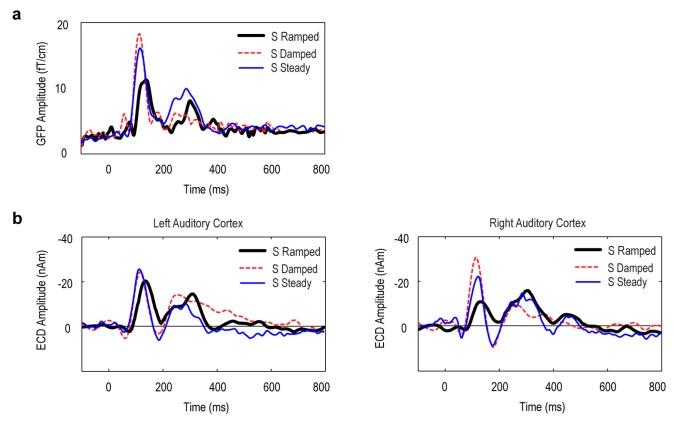
MEG responses for the S steady tone in comparison with the S ramped and S damped stimuli. (**a**) Grand mean global field power data; (**b**) Grand mean equivalent current dipole waveforms showing differences in left and right auditory cortices depending ON response type (ON vs. OFF) and stimulus type (ramped, damped, steady). Repeated measures ANOVA on ECD amplitude data showed significant main effects of response type (F(1, 5) = 72.33, *p* < 0.001), stimulus type (F(2, 10) = 8.92, *p* < 0.05, Greenhouse-Geisser corrected), and a significant three-way interaction between response type, stimulus type and hemisphere (F(2, 10) = 24.75, *p* < 0.01, Greenhouse-Geisser corrected). Repeated measures ANOVA on ECD latency data confirmed significant main effects of response type (ON vs. OFF) (F(1, 5) = 1207.86, *p* < 0.00001), stimulus type (ramped, damped, steady) (F(2, 10) = 13.22, *p* < 0.05, Greenhouse-Geisser corrected), and a three-way interaction between response type, stimulus type and hemisphere (F(2, 10) = 18.54, *p* < 0.01, Greenhouse-Geisser corrected).

As our study was primarily interested in investigating the relationship between perceptual temporal asymmetry and the ON and OFF responses of the ramped and damped stimuli, the comparison with the steady reference tone was not included or discussed in the main text. More statistical results are provided in the appendix to show the comparison of sounds with steady, rising and falling intensity envelopes (Figure A1 and Figure A2).

## 3. Results

### 3.1. Behavioral Data

Behavioral results replicated previous findings. The average d’ scores were above 3 for detecting differences in subjective duration of the ramped and damped stimuli (3.7 for S stimuli and 3.6 for C stimuli). Repeated measures ANOVA results showed a significant main effect of stimulus intensity envelope (ramped vs. damped) (*F*(1, 5) = 94.23, *p* < 0.001), no significant effect of stimulus type (S vs. C stimuli), and no significant interaction between intensity envelope and stimulus type. Each subject judged the ramped sounds to be of longer duration than the damped sounds for both the simple and C stimulus conditions (*p* < 0.00001; Fisher’s Exact Test). On average, the ramped sound was perceived to be longer in 92.5% of the trials for the S stimuli and 91.7% of the trials for the C stimuli. Duration matching results showed that the ramped sound was perceived to be 25.1% longer than the damped sound for the S stimuli (219.2 ms vs. 175.2 ms). Consistent with previous behavioural studies [3], a smaller perceptual bias effect was observed for the C stimuli with the ramped sound perceived to be 14.7% longer than the damped sound (211.7 ms vs. 184.5 ms). 

### 3.2. Global Field Power (GFP) Data

**Figure 2:**
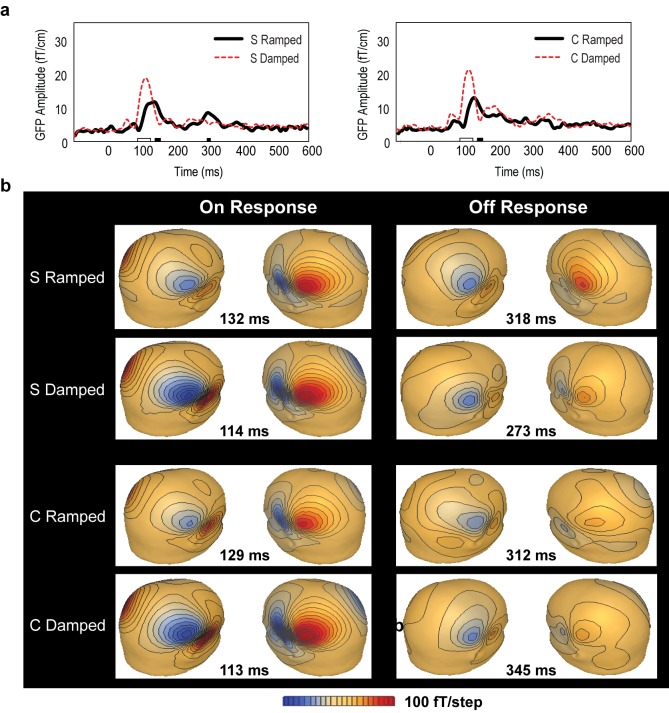
Neuromagnetic responses to the S and C ramped and damped stimuli. (**a**) Grand mean global field power of the MEG responses. Significant differences between the ramped and damped sounds are indicated on the horizontal axis (*p* < 0.01). Intervals with larger amplitudes for damped sounds are marked by white bars on the x-axis, and intervals with larger amplitudes for ramped sounds are marked by black bars. (**b**) Isofield contour maps of the grand mean ON and OFF responses.

Repeated measures ANOVA results for GFP peak amplitude data showed a significant main effect of intensity envelope (ramped vs. damped) (F(1, 5) = 70.1, *p* < 0.001) (Figure 2). There was also a main effect of MEG response type (on vs. off)—the ON response was much larger than the OFF response (F(1, 5) = 94.34, *p* < 0.001). A significant interaction was observed between intensity envelope and MEG response type (F(1, 5) = 68.59, *p* < 0.001). Post-hoc two-tailed *t*-tests confirmed greater ON responses for the damped stimuli than for the ramped stimuli in both S and C stimulus conditions (*p* < 0.01). In the OFF response, the S stimulus condition showed dominance for the ramped sound whereas the C stimulus condition did not (*p* < 0.05). 

Peak latency data showed significantly later ON response for the ramped sounds than the damped sounds (F(1, 5) = 96.8, *p* < 0.00001). Although the GFP data also showed a trend in the OFF response latency in line with our hypothesis, there was no significant difference in the off latency responses between the ramped and damped stimuli in either stimulus condition. These patterns were reflected in the point-to-point comparisons in GFP data (Figure 1) as well as in the isofield contour maps at the MEG sensor level (Figure 2).

### 3.3. Point Source Modelling: Equivalent Current Dipole (ECD) Data

The ECD locations were similar for the ramped and damped sounds in both hemispheres. No significant differences in any of the x, y, and z dimensions were found for the ECD source location between the ramped and damped sounds in either S or C stimulus condition. Repeated measures ANOVA results of the ECD amplitude data showed a significant main effect of intensity envelope (ramped vs. damped) (F(1, 5) = 72.3, *p* < 0.001). There was also a main effect for MEG response type (on vs. off) (F(1, 5) = 116.73, *p* < 0.001).

**Figure 3:**
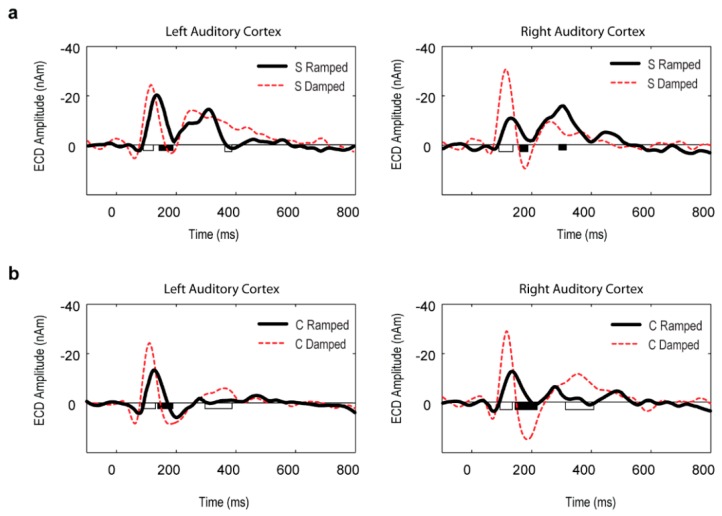
Grand mean equivalent current dipole (ECD) waveform results in the left and right auditory cortices for the ramped and damped sounds in the two stimulus conditions: (**a**) S stimuli; and (**b**) C stimuli. Significant differences between the ramped and damped sounds are indicated on the horizontal axis (*p* < 0.01). Intervals with larger amplitudes for damped sounds are marked by white bars on the X-axis, and intervals with larger amplitudes for ramped sounds are marked by black bars.

The dominant ON responses were observed for the damped sounds relative to the ramped sounds, which was observed in both hemispheres for both stimulus conditions (*p* < 0.01; post-hoc two-tailed *t*-test). In the OFF response, there was a significant interaction between stimulus type (ramped vs. damped), stimulus condition (simple vs. complex) and hemisphere (F(1, 5) = 11.32, *p* < 0.05). The S stimulus condition showed auditory OFF response dominance in the right hemisphere for the ramped sound in comparison with the damped sound (*p* < 0.05; post-hoc two-tailed *t*-test). Like in the GFP data, the C stimulus condition did not show dominance in OFF response in favour of the ramped sound over the damped sound in either hemisphere. Point-to-point comparisons of the ECD source waveforms further confirmed these patterns (Figure 3). 

### 3.4. Distributed Source Modelling: Minimum Norm Estimation (MNE) Data

**Figure 4:**
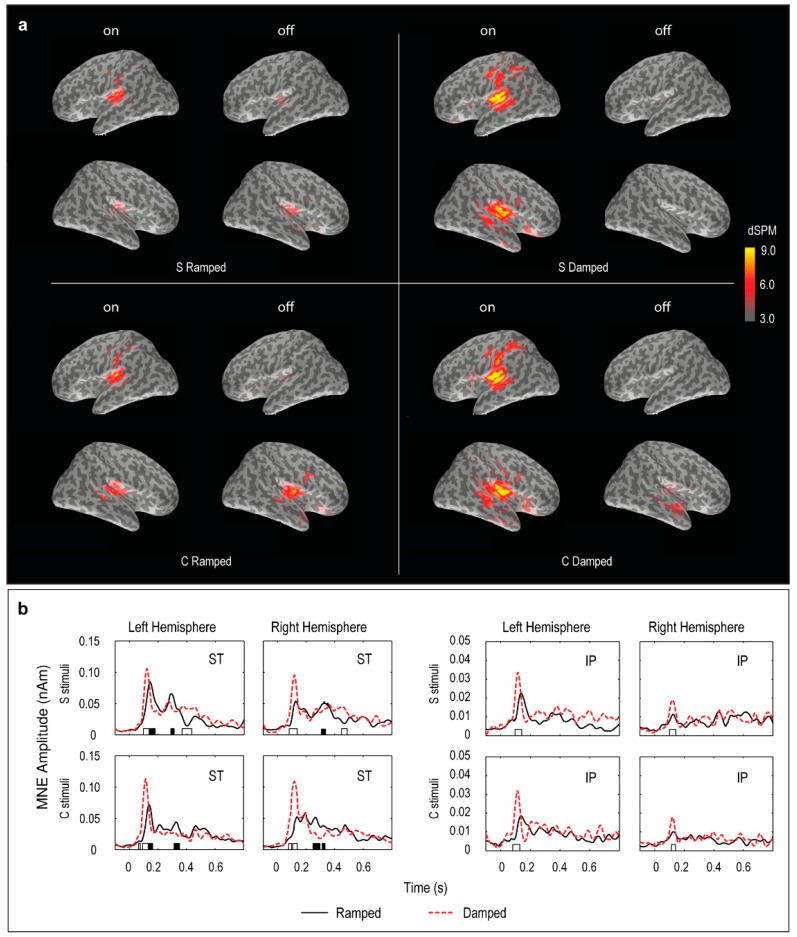
Grand mean MNE waveforms in the two regions of interest (ST and IP). (**a**) ST; superior temporal region. (**b**) IP; inferior parietal region. Bars on x-axis indicate significant differences between the ramped and damped stimuli using the same convention as in Figure 2.

In both S and C stimulus conditions, the MNE data showed two main regions of bilateral activity (superior temporal and inferior parietal) for the ON response (Figure 4). In both superior temporal (ST) and inferior parietal (IP) regions, there was a significant effect of response type (on vs. off) with larger ON response than OFF response (F(1, 5) = 18,28, *p* < 0.01 for ST; F(1, 5) = 18,84, *p* < 0.01 for IP). The ON responses were greater for the damped sounds than those for the ramped sounds in the ST and IP regions, which was further confirmed in separate post-hoc *t*-tests for each hemisphere and each stimulus condition (*p* < 0.05). 

The OFF response showed dominance in favour of the ramped sounds only in the ST region (F(1, 5) = 10.24, *p* < 0.05). There were significant interactions among stimulus condition (S vs. C) and hemisphere (left vs. right) in both the ST (F(1, 5) = 23.31, *p* < 0.01) and IP (F(1, 5) = 6.65, *p* < 0.05) regions, suggesting that stimulus complexity affected the involvement of the auditory areas in the two hemispheres differently in the OFF response. Post-hoc *t*-tests showed OFF response dominance in the right ST region for the ramped sounds in both stimulus conditions (*p* < 0.05). The MNE activity patterns were confirmed in time-point-by-time-point *t*-test for the two regions of interest for each stimulus condition (Figure 4). Consistent with the GFP and ECD results, the point-to-point MNE comparison for the damped and ramped stimuli did not show evidence for our hypothesis that the ramped stimuli would elicit stronger sustained activities following the OFF response in either the ST or IP region in a passive listening condition. 

### 3.5. Percentage Differences in ON and OFF Latencies 

For a direct comparison with previously reported behavioral data, percentage differences in ON and OFF response latencies between ramped and damped stimuli were calculated for all the MEG analysis techniques we used. On average, the ON response latency for the S ramped sound was delayed by 15.8% relative to the S damped sound, which was consistently observed in the GFP peak measure. For the C stimuli, a similar ON response delay of 14.2% was observed. Consistent percentages of delay in the ramped stimuli were found in the ECD analysis (for the S stimuli, 19.8% in the left brain and 14.9% in the right; for the C stimuli, 16.4% in the left brain and 15.2% in the right) as well as in the MNE analysis (for the S stimuli, 18.9% in the left brain and 16.3% in the right; for the C stimuli, 20.7% in the left brain and 18.4% in the right). The grand mean off-minus-on latency value showed a 17.0% longer duration for the S ramped sound than the S damped sound in the GFP peak data. However, the C stimuli did not show such a pattern; in fact, the grand mean off-minus-on latency value was 21.1% shorter in the C ramped sound relative to the C damped sound.

### 3.6. Phase-Locking Factor Data

**Figure 5:**
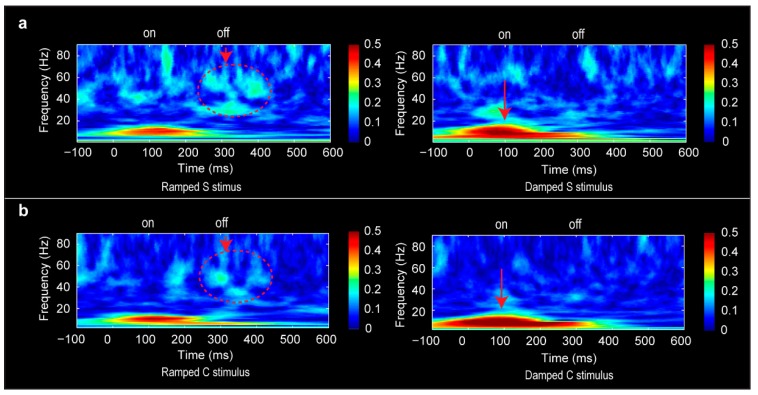
Grand mean phase locking factor results for the S stimulus condition (**a**) and C stimulus condition (**b**). Enhanced gamma band activities associated with the OFF response were marked in dotted circles with an arrow assign for the ramped sounds. Enhanced theta and alpha activities associated with the ON response were indicated by the arrow sign for the damped sounds.

The PLF results confirmed our hypothesis that different neural oscillation patterns mediated the neural coding of rising vs. falling amplitude modulation (Figure 5). In particular, the stronger ON responses for the damped sounds were coupled with stronger PLF at the MEG sensor level in delta (4–7 Hz) (F(1, 5) = 38.10, *p* < 0.001) and alpha (8–14 Hz) (F(1, 5) = 26.36, *p* < 0.01) bands. In contrast, the OFF responses for the ramped sounds were coupled with stronger PLF in gamma (30–70 Hz) in comparison with damped sounds (F(1, 5) = 7.10. *p* < 0.05). Post-hoc tests confirmed these significant differences in PLF in both the S and C stimulus conditions (*p* < 0.05). 

### 3.7. Brain-Behavior Correlates of Temporal Asymmetry

**Figure 6:**
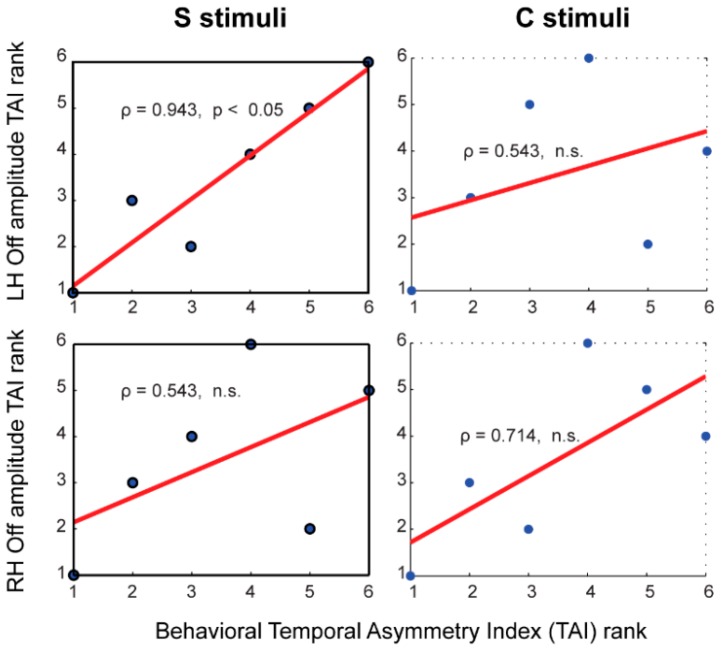
Sample scatter plots of the temporal asymmetry index (TAI) measures with Spearman rank correlation analysis. The OFF response dipole moment (amplitude) was used in calculating the neural TAI scores in the left (LH) and right (RH) hemispheres for the S and C stimuli. Full details of the brain-behavior correlate measures are reported in Table 1.

Spearman rank correlation analysis showed significant results (FDR-corrected) only in the OFF response ECD amplitude of the left auditory cortex for the S stimuli (Table 1 and Figure 6). Even though the ON responses showed a robust effect of ramped vs. damped differences, its temporal asymmetry index (TAI) scores for the ECD data did not show any significant correlations with behavioral data for either the S or the C stimuli. While we observed an overall consistency in percentage differences in the off-minus-on latency for ramped vs. damped sounds with the behavioral percentages of perceived duration, the MEG latency data for ON and OFF responses in the ECD data did not show any significant brain-behavior correlations. The same brain-behavior correlation patterns were also found with the MNE data (Table 1). 

## 4. Discussion

### 4.1. Dominant Auditory ON Response in Favour of Falling Intensity

Despite the limited number of subjects in our study, the analyses using GFP, ECD, MNE and PLF data consistently showed dominant on-N1 response for the damped sounds relative to the ramped sounds regardless of spectral complexity of the stimuli. The point-to-point comparisons for the MEG data clearly demonstrated earlier and larger ON responses for the damped sounds relative to the ramped sounds, and such temporal details were not available from previous functional Magnetic Resonance Imaging (fMRI) data on auditory looming. The MEG source localization results indicated that both left and right auditory cortices contributed to the dominance of the ON response in favour of sounds with falling intensity. This pattern was attributable to the fact that damped sounds involved an abrupt change from silence to the maximum intensity at the stimulus onset. In contrast, the ramped sounds had a much slower intensity change at the onset, effectively reducing and delaying the on-N1 response [80]. 

### 4.2. Limited Evidence for Dominant Auditory OFF Response in Favor of Rising Intensity 

The elicitation of auditory OFF responses was consistent with previous studies that used sounds longer than 100 ms [23,26,27,31]. Consistent with previous fMRI data [81], our MNE results showed right hemisphere dominance for encoding the OFF response to the rising intensity in the auditory stimuli specifically in the superior temporal regionUnlike the auditory ON responses, there was not a uniformly significant effect across all the MEG measures when comparing the OFF responses of the ramped and damped stimuli. It was previously shown that cortical ON responses are encoded more readily and accurately than OFF responses [25]. In our study, the GFP results showed stronger OFF response in favour of the ramped sounds only in the S stimulus condition. This is consistent with previous reports about a reduced asymmetry effect in spectral complex sounds, which is possible from prior learning experience and stimulus familiarity [1,3,10,13,14]. Nevertheless, the MNE and PLF data showed the asymmetry pattern to be biased towards ramped sounds in both S and C stimulus conditions. 

### 4.3. Source Localization for ON and OFF Responses

The source localization results suggest that the ON and OFF auditory evoked response may share the same or overlapping cortical sites for coding abrupt acoustic change as previously suggested [26,27,28]. We did not find statistically different source locations for off and ON responses as has been noted in two physiological studies where OFF responses were found to be either slightly more anterior [35] or slightly more superior than the ON response [32]. As we tested only six subjects with large intersubject variability in the source localization data, subtle differences and small effects might be hard to verify with a small sample. While animal neurophysiology work suggest that different neuron clusters or projection pathways may be responsible for coding ON and OFF response at the levels of thalamus [82,83] and auditory cortex [18], the spatial resolution of our ECD and MNE analysis methods for the human MEG data as implemented in the current study is rather limited and thus may not be able to separate dipole sources for ON and OFF responses that are within the spatial radius of 10 mm [52].

### 4.4. Distinct Neural Oscillations for Rising and Falling Intensities

The PLF data indicate the involvement of distinct neural oscillations for tracking the rising vs. falling intensity modulation direction within the acoustic stimuli. In the present context with a passive listening condition, the dominant PLF for the ON response to the damped sounds was mediated by stronger alpha and theta activity, reflecting new information coding for the abrupt onset [50,51]. As the onset of damped sounds is prone to capture attention/arousal/alertful reaction, there could be differences in involuntary attention to the arrival of the damped vs. ramped sounds mediated by alpha activity, which is known to be influenced by attention [84]. The dominant PLF for the OFF response to the ramped sounds was mediated by gamma activity, reflecting integrated temporal coding of the rising intensity envelope. These data are consistent with animal neurophysiological findings. Recent in vivo patch-clamp whole-cell recordings from the primary auditory cortex of anesthetized rats indicate that the ON and OFF responses are driven by largely non-overlapping sets of synaptic inputs in the auditory cortex [85]. Interestingly, the gamma activities associated with ramped auditory stimuli in our study correspond nicely with MEG data for visual processing of moving vs. stationary stimuli with moving objects eliciting higher gamma oscillations [86]. 

An alternative explanation for the distinct neural oscillation patterns is that phase synchrony may reflect expectation or stimulus predictability [87,88]. One could argue that the offset for a damped sound is more predictable than that for a ramped sound. In addition, our stimulus presentation protocol used variable inter-stimulus intervals (ISIs) between trials, which would make the sound offsets more predictable than sound onsets. Thus, endogenous anticipatory processes may have induced or contributed to differences between the oscillatory responses elicited by the sound offsets and onsets. 

While it is appealing to interpret the phase-locking factor as a measure of oscillatory activity on a phase-resetting account, caution is necessary here as we cannot rule out the traditional additive model for evoked responses [51]. Our PLF data as reported cannot provide conclusive evidence to cleanly separate what might be due to phase-resetting of ongoing oscillations and what might be due to additive evoked response (possibly non-oscillatory) for stimulus coding. In our view, the two models are not necessarily exclusive of each other. Both the phase locking factor and the auditory evoked response measures capture information about neural synchrony across trials in an event-related experimental design. 

### 4.5. Neural Correlates of the Perceptual Temporal Asymmetry 

Spearman correlation analysis revealed significant results only in the amplitude measure of the OFF response in the left auditory cortex for the S stimuli. This phenomenon is consistent with our prediction based on the behavioral literature [1,3,10,13,14]. Behavioral data showed that the ramped sound was 25.1% longer than the damped sound in the S stimulus condition. The subjective duration difference was reduced to 14.7% for the C stimuli. Spectral complexity and sound familiarity of the piano-sound quality of the C stimuli mostly likely reduced the monotonic relationship between the temporal asymmetry index scores for the neural responses obtained in a passive listening condition and behavioral results obtained in an active listening condition. The fact that the ECD and MNE data showed consistent correlation results suggests that both source localization methods provide good estimates of the ON and OFF activities in left and right auditory cortices. The fact that significant brain-behavior correlation was only found in the left hemisphere is consistent with previous reports [38,39], indicating that the OFF response might be more important for coding the perceived duration asymmetry than the ON response. Due to the temporal order of neural responses, there could be more influence from the more recent OFF response relative to the ON response in the internal duration judgment. In this process, the left hemisphere might play a more important role for detecting duration differences whereas the right hemisphere might be more important for frequency discrimination as previous research suggested [89].

Previous studies have shown that there is no simple relationship between duration judgment and differences between the ON and OFF responses [36,37]. Our data provided corroborating evidence that OFF-ON latency differences in any of the MEG measures did not perfectly match perceived duration differences for either the S and C stimuli. This could be partly due to the attentional factor in passive (MEG) vs. active (behavior) listening conditions. 

Compared with the ramped sounds, the ON and OFF responses for the damped sounds showed greater differences in the amount of neural activation. This activation pattern would potentially allow listeners to separate the onset and offset of damped sounds more easily. However, we did not observe either a robust effect of earlier OFF response for the damped sounds or conclusive evidence for stronger sustained neural activity associated with the ramped stimuli. The latency data did not lend direct support to either a diminished perception of damped sounds or an augmented perception of ramped sounds. If listeners typically ignore part of the decay portion for the damped sounds as noted in attentive listening, one would expect the OFF response latency for damped sounds to be earlier than that for the ramped sounds. Similarly, if the ramped offset generated a small amount of persistent sustaining activity, one would expect later OFF response for ramped sounds relative to damped sounds. Nevertheless, it is interesting to note that the percentage of ON response latency delay for the ramped sounds in our two stimulus sets precisely fall within the previously reported range of subjective duration differences for ramped vs. damped sounds when the listeners were instructed to consider all aspects of the sounds [5,7]. 

### 4.6. Limitations and Future Directions

As the S stimuli in our experiment used a linear amplitude envelope and portions of the C stimuli also appeared linear in the sound waveforms, there could be a confounding factor of amplitude acceleration rate differences at the onset and offset of the stimuli when the intensity is expressed on the dB scale. That is, although the waveform shape suggests a linear change in intensity over the tone duration, the velocity of the intensity-change was not constant—there is a brief, high-velocity change at the low-level part of the ramp, and a more gradual one at the high-level part. Previous research has shown that many factors, including intensity level (or audibility), rise time, spectral content, and stimulus duration, jointly influence auditory ON responses. In particular, Biermann and Heil (2000) demonstrated that unlike intensity level and rise time which systematically modulated the auditory ON response, varying acceleration rates of the stimulus envelope at the onset did not affect the auditory ON response in human subjects [43]. While rise time directly affects auditory ON response amplitude and latency [90,91], sound level exerts greater influences than the rising speed [92] and the spectral content of the stimuli is also a very important determinant [93]. Previous studies suggest that the amplitude of the ON response could get smaller with its latency delayed when the amplitude acceleration rate at the sound onset is decreased [94]. Thus if our S stimuli had used a linear envelope on the dB scale, the shallower acceleration rate at the onset of the ramped sound could have resulted in later and smaller ON responses. This hypothetical scenario would then produce even larger on/off differences between the ramped and damped stimuli than what we reported. Since much less is known about how amplitude accelerate rate as well as the other acoustic factors affects the auditory OFF responses [95], further research is necessary to investigate whether this predicted result truly holds with a systematic control of the envelope shape, rise time, level, and duration of the stimuli. 

Due to the limited number of participants and the lack of exact models to formulate hypotheses on the brain-behavior correlates in our study, the statistical results as reported should be interpreted as an exploratory analysis. In particular, one needs to be cautious about issues of low statistical power—a significant pattern with low power from a small sample of six subjects may not necessarily extend to the population level. Previous research on the auditory ON response showed that the adult MEG data (both MEG field measure and dipole model) were highly reliable with a small sample of five subjects in six repeated measures [96]. But it remains unclear whether the OFF response is also highly replicable with a small subject sample. Future studies need to be conducted with a larger sample size to verify the results and test the small or weak effects which a small subject sample may not be able to reveal. 

One potential confounding factor in comparing auditory evoked responses for time-varying sounds is the lack of balance in selective attention to the different stimuli [54,97,98]. Previous behavioral and imaging studies have used an active listening task to demonstrate the selective attentional bias for sounds with rising intensity [15,16,99]. Some researchers have argued for the evolutionary preparation for the perceptual priority and alertness of rising intensity as it is an intrinsic property of an approaching sound source [81,100]. While rising and falling intensities are acoustic patterns associated with approaching and receding sound sources, they are not necessarily reminiscent of auditory motion perception when the ramped and damped stimuli are as short as 200 ms as in our design. In our experimental design using a passive listening condition with a distraction task, the MEG data primarily reflect automatic coding of the physical differences between the stimuli (with the listener presumably taking into account of all aspects of the physical parameters of the ramped and damped stimuli). We were interested in testing predictions related to the two psychophysical accounts by comparing neural coding of rising and falling intensities in auditory stimulation independent of attentional bias. Our results could be interpreted in favour of either theory. The MEG data showed shared cortical sites for the transient ON and OFF responses as well as distinct neural oscillations for coding the dynamic intensity envelopes in the absence of attentional efforts. 

As the current study only used stimuli of 200 ms in a passive listening condition, it remains to be tested whether the asymmetric response patterns are generalizable to stimuli that are shorter or longer than 200 ms and how attention, which has been shown to affect the amount of perceptual asymmetry in behavioural estimation [7], would modulate the neural responses. As our subject sample only included male participants, it also remains to be tested whether the auditory ON and OFF responses would faithfully reflect the sex difference found in behavioural data with females showing a larger effect of perceptual asymmetry [101,102]. 

The preliminary findings of the current exploratory study have important implications for future studies on developmental, cross-linguistic and pathological populations. Auditory ON and OFF responses have been shown to be potential neural markers of brain immaturity in children [32] and cortical dysfunction in adults to assess their ability to extract meaning from dynamic intensity changes in music and spoken language [103]. In dyslexia research, a subgroup of children was found to be linked with a potential deficit in neural discriminatory sensitivity to envelope changes in speech and nonspeech sounds [104]. In a cross-language study, language experience has been shown to play an important role in differentially coding speech sounds in onset and offset positions [105]. Future clinical work and cross-language neurophysiological studies can further test the reliability and diagnostic utility of distinct ON and OFF responses to time-varying sounds with rising versus falling intensity and how they are affected by linguistic experience or pathological conditions. Given that similar looming biases for rising intensity also exist in the visual modality (e.g., [106]) and in multisensory integration [4,99,107,108], future studies with the target populations can also investigate the domain-general mechanisms for the asymmetric ON and OFF responses and potential cross-modal interactions in multisensory integration. 

## 5. Conclusions

In sum, the present study employed MEG techniques to explore and compare the cortical responses to ramped and damped sounds with varying spectral complexity. First, the behavioral results replicated previous studies that ramped sounds were perceived to be longer in subjective duration compared to damped sounds. Second and critically, a robust difference in the ON response between the ramped and damped sounds was observed in the superior temporal and inferior parietal regions, showing a weaker and delayed pattern compared to damped sounds. Unlike the ON responses, the OFF responses did not show a robust effect of dominance in favor of the ramped sounds in either cortical regions. However, consistent effects in neuronal oscillations (alpha and theta activities in the ON response and gamma activities in the OFF response) were observed in relation to the differences between ramped and damped sounds. Finally, a significant correlation was found between the OFF response amplitude in left auditory cortex and behavioral temporal asymmetry for the spectrally simpler stimulus pair. The results indicate distinct asymmetry in ON and OFF responses and trial-by-trial neural synchronization patterns for coding the dynamic intensity changes, which interact with spectral complexity of the auditory stimuli to influence the perceptual bias in favour of rising intensity. These preliminary data have implications for future studies to examine how the auditory system develops such an asymmetry as a function of age and learning experience and whether the absence of asymmetry or abnormal ON and OFF responses can be taken as a biomarker for certain neurological conditions associated with auditory processing deficits. 

## Figures and Tables

**Table 1:** Spearman correlation coefficient (*ρ*) results for brain-behavior correlations in temporal asymmetry index scores, (Q_ramped_ − Q_damped_)/(Q_ramped_ + Q_damped_), for the S and C stimuli (*df* = 5, ECD = equivalent current dipole, MNE = minimum norm estimation, LH = left hemisphere, RH = right hemisphere).

Brain Measures		S Stimuli		C STIMULI	
		ON Response	OFF Response	ON Response	OFF Response
ECD	Amplitude (LH)	*ρ* = 0.429	*ρ =* 0.943 *	*ρ* = 0.257	*ρ* = 0.371
	Latency (LH)	*ρ* = −0.551	*ρ* = 0.086	*ρ =* 0.714	*ρ =* −0.086
	Amplitude (RH)	*ρ =* 0.143	*ρ =* 0.543	*ρ =* 0.371	*ρ =* 0.714
	Latency (RH)	*ρ =* −0.577	*ρ =* −0.086	*ρ =* 0.377	*ρ =* −0.029
MNE	Amplitude (LH)	*ρ =* 0.257	*ρ =* 0.829 *	*ρ =* 0.200	*ρ =* 0.086
	Latency (LH)	*ρ =* 0.200	*ρ =* 0.600	*ρ =* 0.489	*ρ =* 0.200
	Amplitude (RH)	*ρ =* 0.657	*ρ =* 0.714	*ρ =* 0.257	*ρ =* 0.486
	Latency (RH)	*ρ =* 0.029	*ρ =* 0.493	*ρ =* 0.086	*ρ =* 0.543

Significant correlations are indicated with a star sign (* stands for *p* < 0.05, FDR-corrected). The significance of the correlational coefficients in bold italics obtained from our limited number of data points was also confirmed in the resampling bootstrap procedure of up to 1000 samples using the robust correlation toolbox [78].

## Abbreviations

The following abbreviations are used in this manuscript:

MEG:	Magnetoencephalography
EEG:	Electroencephalography
EOG:	Electro-oculogram
ECD:	Equivalent current dipole
MNE:	Minimum norm estimation
dSPM:	Dynamic Statistical Parametric Mapping
PLF:	Phase locking factor
LH:	Left hemisphere
RH:	Right hemisphere
ROI:	Region of interest
ST:	Superior temporal
IP:	Inferior parietal
RMS:	Root mean square
TAI:	Temporal asymmetry index
MRI:	Magnetic Resonance Imaging
fMRI:	Functional Magnetic Resonance Imaging
TR:	Repetition time
FA:	Flip angle
TE:	Echo time
NEX:	Number of excitations
HPI:	Head position indicator
ANOVA:	Analysis of Variance
FDR:	False discovery rate
ISI:	Inter-stimulus interval
